# Alternative Management Systems of Beef Cattle Manure for Reducing Nitrogen Loadings: A Case-Study Approach

**DOI:** 10.3390/ani11020574

**Published:** 2021-02-23

**Authors:** Yubin Wang, Suraj Ghimire, Jingjing Wang, Renjie Dong, Qian Li

**Affiliations:** 1 College of Economics and Management, China Agricultural University, Beijing 100083, China; wyb@cau.edu.cn; 2 Department of Economics, University of New Mexico, Albuquerque, NM 87131, USA; ghimires@unm.edu (S.G.); wangj@unm.edu (J.W.); 3 National Center of International Joint Research of Bioenergy Science and Technology, College of Engineering, China Agricultural University, Beijing 100083, China; rjdong@cau.edu.cn; 4 College of Economics, Beijing Technology and Business University, Beijing 100048, China

**Keywords:** beef cattle, sustainable manure management, recycling systems, nitrogen loadings

## Abstract

**Simple Summary:**

Livestock manure is one of the primary sources of agricultural nonpoint source pollution and poses a great threat to the environment and human health. Sustainable management of manure via recycling is an effective means to tackle the problem. Based on field interviews in China, four alternative manure management systems were investigated: Compost-based systems, product-based systems, substrate-based systems, and biogas-based systems. For each system, the reasons of emergence, success factors, risk factors, operation mechanism, scalability, key elements, and environmental effects were discussed. Results showed that the adoption of a system is driven by various factors and market-oriented operation is the dominant operation mechanism of all the manure management systems. Compared to direct application of manure to croplands, all the manure management systems can reduce nitrogen loadings from livestock farms and lower their environmental effects. Specifically, biogas-based systems can reduce nitrogen loadings to the greatest extent, followed by product-based systems and substrate-based systems, and then by compost-based systems. Integrated management of manure with mixed recycling systems is imperative for reducing its environmental effects, which can benefit from the increasing role of third-party entities in manure recycling. Policy implications were also discussed.

**Abstract:**

Livestock manure is one of the main sources of agricultural nonpoint source pollution and poses a great threat to the environment and human health. Sustainable management of manure via recycling is an effective means to tackle the problem. Based on field interviews in China, multiple case studies were employed to investigate alternative manure management systems. Four conclusions arose. First, compost-based systems, product-based systems, substrate-based systems, and biogas-based systems were identified as four main types of manure management systems, with each possessing its success factors and risk factors. The adoption of a system was driven by various factors. Second, market-oriented operation was the dominant operation mechanism of all the manure management systems. Third, compared to direct application of manure to croplands, all the four manure management systems could reduce nitrogen loadings from livestock farms and lower their environmental effects. Among the systems, biogas-based systems could reduce nitrogen loadings to the greatest extent, followed by product-based systems and substrate-based systems, and then by compost-based systems. Lastly, integrated management of manure with mixed recycling systems is imperative for reducing its environmental effects, which can benefit from the increasing role of third-party entities in manure recycling. Policy implications were also discussed.

## 1. Introduction

With the improvement of human living standards and changes in human diet structure, the livestock sector has proliferated worldwide, leading to considerable environmental pollution caused by livestock manure [1]. Since the beginning of the 21st century, over 30 million tons of livestock manure (N content) have been produced worldwide annually, a number that keeps growing [2]. For global food security and food production sustainability, it is essential to improve livestock operations’ environmental performance [3,4,5], necessitating manure management strategies with minimal environmental impact [6]. In the last three decades, China has experienced a substantial transformation in its livestock industry, making it the world’s largest producer and consumer of livestock products. This has profoundly affected its domestic and global food provision, resource use, nitrogen and phosphorus cycles, and greenhouse gas emissions [7]. China is now the world’s leading livestock manure generator, producing about 18.22% of the world’s livestock manure (N content) in 2017 [2]. The China Handbook of Manure Production [8] divides China into six regions, each with their unique manure production paradigms. Table 1 shows the total amount of livestock manure produced in different provinces of China in 2017, calculated based on the livestock population (including pigs, cattle, sheep, horses, donkeys, mules, and rabbits). In 2017, 959.36 million tons of manure was generated in China, of which Inner Mongolia, Sichuan, and Xinjiang, the top three manure producing provinces, produced 11.03%, 8.34%, and 6.8% of the total, respectively. Unsurprisingly, animal husbandry is a primary enterprise of these provinces. Inner Mongolia and Xinjiang also have substantive pastoral areas. The principal livestock of Inner Mongolia includes beef cattle, cow, and sheep, Sichuan mainly has pigs and beef cattle, and Xinjiang has a large sheep population.

From a regional perspective, the southwest region is the leading producer of manure in China. In 2017, the amount of manure produced in this region was 223.83 million tons, nearly a quarter of the country’s total. The south-central region, which consists of six provinces and two special administrative regions, ranked second in manure production, accounting for about 21.18% of the national total. Henan province is the largest contributor of manure in the southwest region, producing 52.38 million tons. East China produced the least amount of manure, accounting for only 9.03% of the national aggregate, which was less than Inner Mongolia province’s total. Because of the industrial development in East China, especially Shanghai and Zhejiang provinces, the practice of animal husbandry is declining year after year. In terms of manure produced, these two provinces ranked first and third from the bottom.

China faces a significant challenge in sustainable manure management [11], with worldwide attention drawn towards the country [12]. Developed countries with a trend towards concentrated animal feeding operations face similar challenges, such as the dairy industry’s manure management scenario in the United States [13,14,15]. Beef cattle, however, exert more significant environmental pressure due to the higher excretion rate of manure. Therefore, sustainable management of beef cattle manure has become a vital research topic.

Land application of manure as an amendment has multiple benefits. Applying manure to the soil, in the long run, can improve the soil structure, increasing water retention capacity, soil root penetration, and microorganism activities, and thereby increasing the ability of crops to absorb nutrients [16,17,18]. Replacing synthetic fertilizers with manure can provide an additional supply of nutrients such as potassium, magnesium, copper, and zinc, leading to greater crop yields [19,20,21,22]. This reduces the need for synthetic fertilizers, offering a direct monetary incentive for the farmers. A fraction of the N available as organic N in cattle manure is slowly released over time after mineralization. This results in an increased total N uptake, improved nitrogen use efficiency, and growth in crop yield [21,23]. Land application of manure disperses the nutrients and other byproducts of livestock operations to a wider area, reducing the localized environmental externalities such as the contamination of water sources, obnoxious odor, and pathogen loading. There is a strong correlation between the soil’s organic matter content and its quality [24]. A meta-analysis of available research from more than 130 global observations found that regular application of livestock manure explained more than 50% of the variability in soil organic carbon stock than mineral fertilized or unfertilized soils [25]. A similar meta-analysis of the studies done in China comparing the benefits and downsides of synthetic fertilizer and livestock manure towards crop productivity found that partial substitution of synthetic fertilizers with manure increased the yield by 6.6 and 3.3 percentages for upland crops and paddy rice, respectively [26]. Livestock manure is preferred over other organic fertilizer sources due to its shorter decomposition period [27,28]. However, livestock manure’s efficacy depends on various factors such as the optimal mix with synthetic fertilizer, pH level of the soil, and experimental duration. In addition to these clear benefits, there are some drawbacks of substituting synthetic fertilizers with livestock manure [25]. The total substitution of synthetic fertilizer with manure had, in fact, a negative impact on crop yields [25].

Adding manure to the soil changes its chemical and physical properties. The changes in chemical properties include the change in concentration of nutrients, trace element profile, and pH level. The alteration in the soil’s physical properties such as permeability, hydraulic conductivity, aggregation, and bulk density can affect nutrient and water movement within the soil profile [29,30]. The increase in the concentration of highly soluble nitrate-nitrogen and phosphate ions, as well as the increase in porosity of the soil, can result in the increased transportation of nutrients to surface water streams via drainage which can be exacerbated by precipitation events and flooding [31]. Based on the water table’s depth and soil properties, these nutrients can also be leached into underground aquifers. The enrichment of surface water can result in excessive growth of algal and aquatic plant populations, resulting in eutrophication. This can result in a massive decline in aquatic biodiversity and directly impact human health with the ingestion or exposure to the water containing harmful algal toxins. The volatilization of ammonia from the manure during excretion, collection, storage, treatment, and application is a significant concern that needs to be addressed. The volatilization of ammonia pollutes the surrounding air and pollutes waterways, and negatively affects residents’ livelihood via acid rain deposition. Furthermore, the lost ammonia is a direct loss of nitrogen fertilizer, reducing agricultural productivity and profitability. The use of a shallow injector and band spreader to spread liquid manure can substantially reduce ammonia volatilization compared to broadcast surface spreading [32]. Additionally, proper design of feedlots, covered storage system, and controlling the amount and timing of manure application can help mitigate nutrient buildup and, subsequently, waterways’ pollution [33,34,35]. Solid–liquid separation also effectively strips certain nutrients and solids from liquid waste, which can then be a more stable form of fertilizer or used as bedding [35].

Land application is still a major way to utilize livestock manure in developing countries [36], e.g., in South Africa, livestock manure is mostly left in the pasture or paddocks or managed as drylots [37]. Manure can be returned to land either directly or after composting and should be applied based on manure characteristics, soil types, and agronomic requirements of crops [11]. However, with the development of intensive animal farming and the specialization of livestock and crop production, the practice of returning manure directly to cropland is becoming more problematic. The main problem is insufficient cropland to spread manure due to excess nutrients associated with concentrated animal feeding operations [38,39,40].

Biogas production, categorized by anaerobic digestion (AD) treatments, refers to the degradation of organic materials by microorganisms in the absence of oxygen to produce biogas [41]. Biogas derived from AD of animal, human, and other organic wastes has a long history of use as a source of household energy in developing countries [42]. Its utilization at a larger scale is emerging in developed countries. Recycling manure for biogas production is both environmentally beneficial as a sustainable way to dispose of manure and economically valuable as a source of renewable energy and biofertilizer [43]. The adoption of biogas technology depends on various environmental, economic, technical, and social factors [44]. In France, AD biogas production is estimated to expand and reach the European target of 20% of energy from renewable sources [38]. In China, the proportion of livestock manure used in biogas production increased rapidly from 2005–2010, benefiting from biogas project’s national promotion [45]. An extensive literature has focused on the cost–benefit analysis or feasibility analysis of manure management strategies, including AD, mostly using the life-cycle assessment for individual farms [46,47]. Despite the high upfront costs of biogas produced by AD, it could be a profitable strategy given subsidies and tax credits for renewable energy projects [14,48]. Several studies compare different biogas digesters and find that the fixed biogas digester, especially the small version, is the most economically and environmentally feasible [49]. However, some studies find the opposite: Farmers cannot afford to install and maintain biogas digesters, rendering the commercialization of biogas projects mostly unfeasible [50].

Various novel methods have been developed over the years in response to conventional manure management practices’ limitations. Whalen et al. explored novel practices and smart technologies to optimize the benefit of using manure as an N supplement in cold, humid temperate regions [51]. The study examined sensor technologies with advanced decision-making algorithms to improve manure handling and application to optimize manure’s N fertilizer value. These smart systems use a network of wireless ammonia-detecting sensors at various lagoon manure locations. The sensors trigger automated responses by adding aluminum chloride, alum, sulfuric acid, or ferric chloride to lower the pH level, coagulate, precipitate, or flocculate the wastewater. This process reduces gaseous ammonia losses by absorbing the ammonium ions and changing their chemical properties. The sensors can also activate the automatic placement or removal of the lagoon covers. Thermocouples and moisture probe can automatically turn and hydrate the manure stockpile, and bulking agents stimulate the decomposition process.

Manure side-dressing is another practice that has proven effective in improving the nitrogen use efficacy of the fertilizers [51]. A smart system can determine the optimal N application rate and the ratio of side-dressing manure during the growing season. Side-dressing is the practice of spreading and incorporating manure besides the row of annual crops. Machine learning algorithms are used to calculate the variable manure application rates. Some other technologies effective in recovering the nutrients from manure and wastewater include microbial-based technologies [31,52]. This involves growing algae or plants in wastewaters, which would recover excess nutrients to be recycled later. Other practices include oxidation ponds, facultative lagoons, constructed wetlands, storage ponds, and composting [31,53]. Superheated steam drying technology is also an alternative manure management method in which cow manure is rapidly treated with hot steam for use as an alternative fuel. The results show that this method has 95% less eutrophication potential compared to direct field application [54].

Despite numerous farm-level studies evaluating the technical feasibility, economic benefits, and/or environmental impacts of alternative manure management practices for individual farms [55,56,57,58,59,60,61,62,63], few studies have comprehensively investigated the critical factors affecting the choice and performance of such systems. The main objective of this study was to explore and understand the determining factors, and critical success and risk factors for alternative manure management systems. When the boundaries of a phenomenon are unclear, and there is no control over behavioral events, a case-study approach is desired and can be used to identify a set of critical variables for future quantitative investigation [64,65]. In this study, the boundaries—factors that may significantly influence the choice and performance of alternative manure management systems—were still relatively vague. Furthermore, since manure management practices differ from sector to sector and from country to country, it was desirable to focus on one type of livestock manure in one country before moving onto cross-sector and cross-country studies. To this end, a single research design focusing on the management of beef cattle manure in China was chosen.

Typical cases were selected to analyze the reasons of emergence, success factors, risk factors, operation mechanism, scalability, key elements, and environmental effects of each type of manure management system. The results were expected to provide improved information and policy recommendations for livestock manure management in China as well as in other countries or regions facing similar challenges of manure disposal.

## 2. Materials and Methods

### 2.1. Policy Background

The intensification of the livestock industry and the environmental externalities due to manure mismanagement have become a major public policy concern all over the world. Most developed countries have stepped up to address these concerns by developing and revisiting policies pertaining to the various stages of manure handling, storage, application, and treatment. Developing countries either do not have a comprehensive manure management framework or have contradictory policies and non-compliant agents to enforce the existing manure legislation [66]. Table 2 provides an overview of the manure policy frameworks across 14 countries and regions of the world [66,67,68].

The major policy support mechanism for livestock manure management in China is via policies for renewable energy production. For reasons ranging from air pollution to energy security, China has been developing renewable energy for years [69]. According to the National Energy Administration of China, by the end of 2019, China’s renewable energy power generation capacity reached 794 million kilowatts, accounting for about 39.5% of all electricity’s installed capacity, and renewable energy is expected to become the main incremental source of energy consumption [70]. The rapid development of renewable energy in China benefits from the strong support of national policies. In 2005, China promulgated the “Renewable Energy Law,” which specifies the development direction of renewable energy in terms of industrial guidance and technical support, promotion and application, price management and fee compensation, economic incentives, and supervision measures in the form of law. After that, a series of detailed implementation policies were introduced. For example, in 2012, the “Renewable Energy Power Generation Quota Management Measures” were introduced to implement a renewable energy power quota system and clarify the obligation of power generation companies, grid companies, and local government. After 2015, given the difficulty of renewable energy consumption, a pilot project of renewable energy consumption was implemented to ensure renewable energy’s full guaranteed purchase. According to recent statistics as of 2016, China has issued more than 100 policies to promote the development of renewable energy, including the renewable energy grid subsidy, renewable energy power quota system and green power certificate, promotion of renewable energy technology R&D, technological progress policies, and policies to promote renewable energy electricity consumption [71]. It has formed a policy support system mainly based on renewable energy price subsidies and cost-sharing.

Biogas is an important part of renewable energy. China began to promote rural household biogas projects on a large scale in the 1990s and subsidized their construction for rural households and small farms through the “Rural Small Public Welfare Subsidy.” Since the 21st century, a series of laws, regulations, and policies has been issued, focused on supporting large-scale biogas production, such as supporting specialized enterprises and large-scale farms to build large-scale biogas projects with a total volume of 500 cubic meters or more of anaerobic digestion equipment. In terms of raw material utilization, for the utilization of straw and other raw materials and raw material bases, business entities can benefit from fiscal and taxation support policies related to biogas energy. In terms of biogas construction, rural household biogas construction is included in the scope of national debt fund support, large-scale biogas project construction is subsidized, and preferential policies are given in terms of land, electricity, and taxation. In terms of equipment, biogas production, purification, transformation, and other related equipment are included in the agricultural machinery purchase subsidy list. In terms of terminal products, subsidies are provided for terminal products such as biogas and methane fertilizer. The subsidy standard is 0.25 RMB per kilowatt-hour, and the subsidy time limit is 15 years. For the development of the biogas industry, China has developed a complete supporting policy system covering the sources of raw materials, technological development, engineering construction, and the use of terminal products.

China has integrated financial funds to support the upgrading of farming facilities related to manure treatment, construction of manure storage yard, sewage storage pool, anaerobic fermentation pool, oxidation pond, and advanced sewage treatment, composting, and fermentation facilities. Local governments are encouraged to subsidize the manure management equipment as much as possible with the central government’s agricultural machinery purchase subsidy funds. To support the replacement of chemical fertilizer with organic fertilizer, taxpayers who produce, sell, wholesale, and retail organic fertilizer products are exempt from value-added tax. For the energy utilization of manure, policies of the on-grid benchmark electricity price for biogas power generation and the full guaranteed purchase of electricity generated by biogas and the immediate exemption policy of value-added tax on biogas were implemented. For large-scale biogas projects, the central subsidy is 1500 RMB per cubic meter of anaerobic digestion unit volume, and cannot exceed 30 million RMB per project, and the proportion of subsidy cannot be more than 35% of the project investment. The construction of large-scale natural gas projects, biogas projects, organic fertilizer plants, and centralized livestock manure treatment centers will be provided preferential access to land and electricity.

### 2.2. Manure Management System

Existing management systems of cattle manure in China can be categorized into four types: (1) Compost-based systems, where manure is simply composted and then applied to land as organic fertilizers; (2) product-based systems, where manure is deep-processed into commercial organic fertilizer with specialized equipment; (3) substrate-based systems, where manure is used as substrates to produce other agricultural commodities, and (4) biogas-based systems, where manure is used to produce biogas.

In compost-based systems, cattle manure is transported and applied to crop fields directly upon collection or after simple composting and fermentation, which to a certain extent, can reduce the use of chemical fertilizers. This type of manure management system is characterized by low technical and capital requirements. Although labor-intensive, it is the preferred cattle manure recycling system, especially favored by small- and medium-scaled farms, typically owned and operated in rural settings. It is currently the dominating approach of recycling cattle manure and the most critical organic fertilizer resource in the rural areas of developing countries [72]. However, farms adopting this system are often under the pressure of environmental regulations.

In product-based systems, cattle manure is collected and processed into granular or powdered organic fertilizers, which are then marketed and sold to a broader group of consumers in larger markets. This type of manure management system requires extensive capital and technology investments to establish manure processing facilities. As a result, it is applicable only when the cattle farming scale reaches a certain level, and the larger the farming scale, the more feasible it is to adopt. Farms face much less pressure from environmental regulators when product-based systems are adopted.

In substrate-based systems, cattle manure is mixed with other organic waste and processed into substrates to produce other agricultural commodities. Substrates are commonly used in fungiculture (e.g., mushroom farming) or vermiculture (e.g., raising earthworms) and produced in two ways. One way is to add cattle manure either directly or after being fermented to raw substrates. The other way is to use a mix of organic waste (e.g., straws, sawdust, rice husks, mushroom residues, and peanut shells) as padding materials in the cattle feedlots to produce a semi-decomposed mixture through cattle excretions and trampling over time. The mixture is then collected as a substrate to produce fungi or earthworms [73]. The substrate-based systems are integrated agricultural systems capable of producing multiple commodities. Under such systems, farms face low pressure from environmental regulations and benefit from economies of scope.

In biogas-based systems, cattle manure is collected into biogas digesters to produce biogas through anaerobic fermentation. Residues and slurries from the digestion can be applied to crop fields for nutrient recycling. Because cattle manure’s energy density is not high enough to meet the biogas production requirement, other organic wastes like straws, domestic garbage, and other waste generated in livestock farms are added to enhance biogas production. Biogas-based systems typically require high upfront costs and are challenging to maintain stable operation for the long run. Therefore, it may be difficult for small-scale cattle farms to adopt biogas-based manure management systems. It is used at the farm scale in most Asian countries [74].

### 2.3. Sample Descriptions

**Figure 1:**
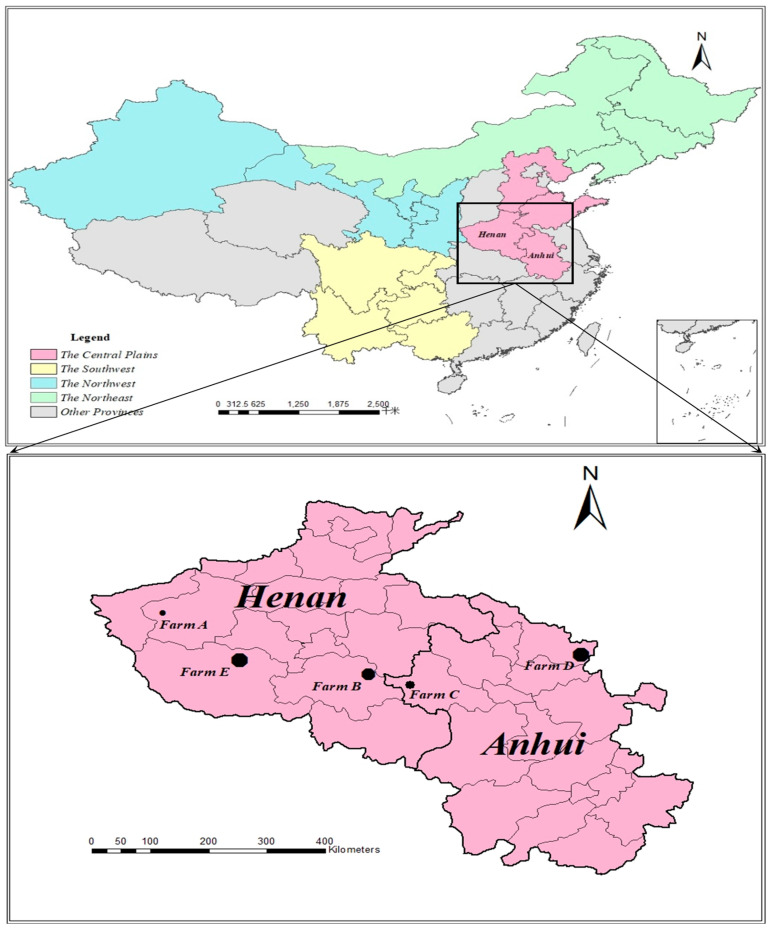
Locations of case-study farms in the Central Plain of China.

Multiple case studies of cattle farms with different sizes and scopes are employed to investigate the characteristics of and identify the four manure management system’s critical success and risk factors. As shown in the upper panel of Figure 1, the Central Plain is where China’s cattle industry originated and remains the largest beef-producing region. In 2018, the Central Plain region’s beef production reached 1.76 million tons, accounting for 27.39% of China’s total beef output [40]. The Central Plain hosts cattle farms of various scales and is ranked first for large-scale farms in the nation. Cattle farms with annual slaughter between 500–999 heads and above 1000 heads in the region account for 30.51% and 30.28% [75], respectively, of the national total. There are two major types of cattle farms in the region: Cow-calf-cattle farms and stocker-finishing farms. These farms can use either grazing or confined-feeding, or a hybrid grazing and confined-feeding system.

Five representative cattle farms were selected from five counties across two provinces (Henan and Anhui) in the Central Plain, as shown in the lower panel of Figure 1. Henan province and Anhui province are two adjacent provinces with similar locations, resources, markets, local institutions, and cattle farming culture. During July–August 2018, semi-structured in-person interviews were conducted at each farm to understand their operating status and manure management system. The interview time averaged 55 min. After each interview, onsite visits to the farms were arranged to get a first-hand understanding of their manure management system, which involved follow-up interview questions to obtain detailed information.

Table 3 summarizes the characteristics of the sample cattle farms based on the data from the interviews. Out of the five farms, two were cow–calf cattle farms, and three were stocker-finishing farms. Cattle farms in China are officially divided into six categories based on the annual slaughter numbers. The categories 1–9 heads, 10–49 heads, 50–99 heads, 100–499 heads, 500–999 heads, and over 1000 heads, respectively, accounted for 95.39%, 3.76%, 0.60%, 0.20%, 0.03%, and 0.01% of all cattle farms in 2019 [75]. The annual number of cattle slaughters in our sample farms ranged from 25 to 1000 heads, representing various cattle farm categories. The sample farm’s annual profit ranged from 1500–6000 RMB/head (i.e., 228–912 $/head) and had a nonlinear relationship with either farm type or farm scale. Farms A, B, C, and D incorporated each of the four types of manure management systems, while Farm E comprised a combination of all four systems. In sum, the five sample cattle farms ensured good representativeness of the research purport (see Appendix A).

### 2.4. Methods

The China Handbook of Manure Production provides the manure and nitrogen excretion coefficient of different livestock in six regions of China as discussed in Table 1 [8]. Based on this, beef cattle’s nitrogen excretion coefficient from the sample area was 65.93 g/head-day (denoted by λ), representing the theoretical amount of nitrogen produced by each beef cattle every day. Therefore, λ was set as the reference value of beef cattle’s nitrogen emission in this study area, and nitrogen loadings of 100% were assumed under the reference scenario of no treatment of manure. Then the nitrogen loadings rate (denoted by *NR*) of the five cases in this research were evaluated by technical experts of the research group. According to the theoretical value of nitrogen loadings and nitrogen loadings rates, the practical nitrogen loadings (denoted by *NL*), nitrogen loadings reduction rates (denoted by *NRR*), and nitrogen loadings reduction values (denoted by *NLR*) were calculated as illustrated in the following equations.

*NL* = *λ* × *NR* (1)

*NRR* = 1 − *NR* (2)

*NLR* = *λ* × (1 − *NR*) (3)

## 3. Results

### 3.1. Individual Case Studies

#### 3.1.1. Compost-Based Systems: Farm A

**Figure 2:**
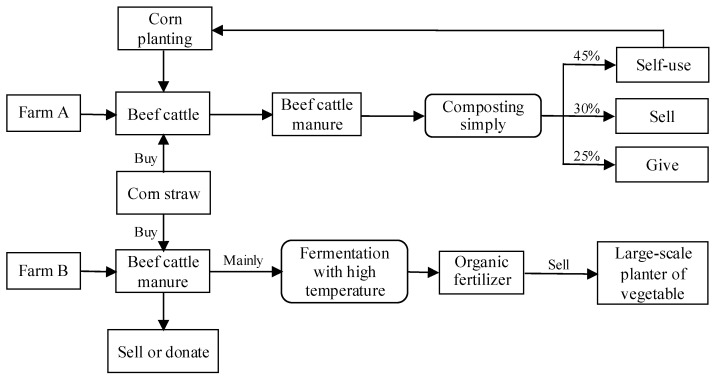
Compost-based systems and product-based systems.

Farm A used a compost-based system to recycle manure, as shown in Figure 2. The economic benefits of manure recycling were the driving force of its management system. Due to financial constraints, the farm would prefer to keep using the compost-based system for manure management for the future. In terms of scale and the business system, Farm A represented the small- and medium-sized cattle farms in China. The legislation and public concern regarding livestock production’s environmental impact have increased the pressure on farmers to reduce environmental pollution [76]. Thus, many beef cattle farms (households) have actively or passively built manure yards and sewage pools. These rudimentary facilities and equipment enhance the traditional way of manure recycling to crop farming. Under the compost-based manure system, the farm can recycle manure through self-use, donation, and sale to nearby farms. The mixed crop-livestock systems are built in the farm, which is considered beneficial for sustainable agriculture [77]. However, the organic fertilizer products cover a small local market with a low commercialization rate.

#### 3.1.2. Product-Based Systems: Farm B

Farm B used a product-based system to recycle manure, as shown in the lower part of Figure 2. Farm B adopted this manure management system because of the two stable contractual relationships with nearby farmers. First, the farm acquired corn silage and straws from nearby farmers, as the Central Plain is also a prominent grain-producing region of China with rich corn straw resources. Contracting corn silage enables a cheap and stable supply of cattle forage, which effectively lowers the cost of cattle farming and stabilizes the farming scale. Contracting corn straws ensures the supply of supplemental raw materials for the manure processing equipment. Second, the farm maintained a close relationship with nearby vegetable growers as a stable marketing channel. The challenge of maintaining product-based systems in the region is not the manure processing technology, which is relatively mature and commercialized, but the selling of organic fertilizers produced from such systems. Crop growers in China typically have a lower recognition of the organic fertilizers produced from cattle manure, so their willingness to purchase it is low. It is not uncommon for manure processing firms to mainly rely on the government purchase of organic fertilizers for product marketing where a viable market has not been established [72]. In sum, both the stable supply of raw materials and reliable sales channels are essential for establishing and maintaining product-based systems for manure recycling.

#### 3.1.3. Substrate-Based Systems: Farm C

**Figure 3:**
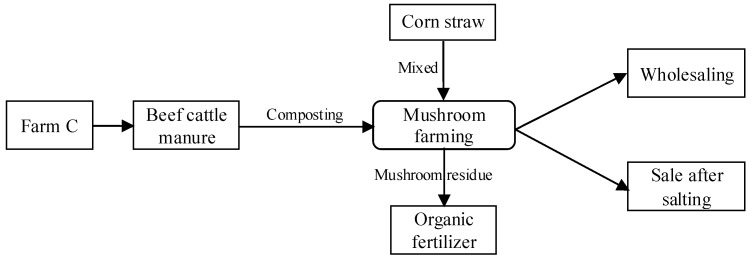
Substrate-based systems with fungiculture.

Farm C used a substrate-based system to recycle manure via fungiculture, as shown in Figure 3. All the manure generated on the farm was used to produce the substrate for mushrooms. The viability of the farm’s substrate-based system depended on its stable mushroom business, which the farm had developed by investing in equipment and facilities to secure the production, storage, and transportation of mushrooms. The farm employed two different cultivation methods of mushrooms: Ground greenhouse planting and an industrialized planting workshop. Under the context of expensive and challenging land expansion, industrialized planting workshops are becoming popular.

However, the substrate-based system has three potential barriers for new businesses. First, this type of manure management system is capital intensive. In total, the farm financed 6 million RMB to construct the mushroom cultivation facilities. Second, sales of mushroom products can face high price uncertainty in local agricultural markets. Lastly, if not contracted, nearby farms can become unreliable sources for procuring manure. It might also pose a potential safety and health hazard to the farm itself due to pathogens contained in the external manure.

#### 3.1.4. Substrate-Based Systems: Farm D

**Figure 4:**
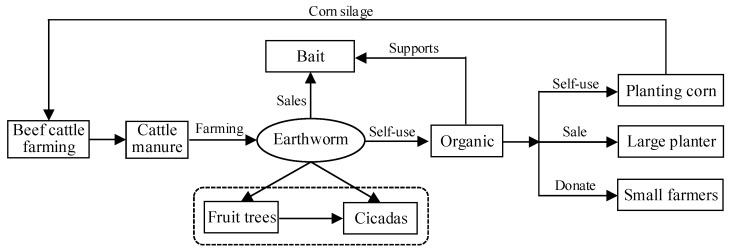
Substrate-based systems with vermiculture.

Farm D used a substrate-based system to recycle manure via vermiculture, as shown in Figure 4. Earthworm farming is a popular substrate-based system for cattle manure recycling. The average profit of earthworm farming can be up to 20,000 RMB/hectare, excluding land rent, labor cost, baby worm introduction, and other production costs. Furthermore, earthworms can generate 400–500 kg of premiere organic fertilizer per ton of cattle manure. In addition, the ridges of worm lands can be used for inter-planting fruit trees and inter-farming cicadas. Therefore, farm owners can realize multiple benefits by adopting an integrated planting–farming cycle. The farm also brought positive spillover effects to the local economy. First, it motivated and provided technical assistance to nearby farmers for earthworm farming. Second, it led to the establishment of a manure treatment center in the county.

Similar to Farm B, Farm D also had reliable raw material supply in its upstream supply chain and stable product demand in its downstream supply chain. The downstream products possessed strong uniqueness and were directly supplied to wholesalers. If expanded to a particular scale, it is even possible for the farm to collaborate with pharmaceutical companies for earthworm supply. Thus, stable contractual relationships and sales channels were two critical factors for successfully executing substrate-based manure management systems with a high-profit margin. Moreover, the farm took the lead in building a manure treatment center to assist other farmers in treating manure. The initiative received support from the government and benefitted from economies of scale in manure management. As shown in Figure 4, the substrate-based system with vermiculture is the core of the planting-farming circular economy, expanding outward and extending the profit chain.

#### 3.1.5. Biogas-Based Systems: Farm E

**Figure 5:**
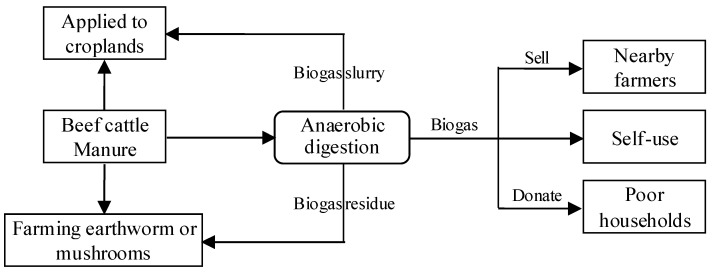
Mixed systems of manure management.

Farm E mainly used a biogas-based system, which was a mix of all the four types of manure management systems (compost, product, biogas, and substrate-based), as shown in Figure 5. The biogas slurry produced was transported to crop fields through pipelines, and the biogas residues were directly applied to crop fields or utilized as the substrate for mushroom and earthworm farming. The mixed biogas–substrate–fertilizer system alleviates the pressure of environmental regulation and generates considerable economic benefits.

The farm’s biogas-based system of manure management benefitted from its large scale and long-term development plan. The biogas production was not limited to self-use, and the farm envisioned supplying surrounding farmers, households, and firms with large-scale biogas production. Besides the biogas power generation, the farm also intended to engage in biogas purification and biogas connection into natural gas pipeline networks. The adoption of a biogas-based system was based on two factors. First, the farm itself had a large scale, which ensured a large amount of manure as a fixed supply of raw materials for biogas production. Second, the farm maintained semi-contractual relationships with nearby smaller cattle farms under an agricultural cooperative, which provided another relatively stable cattle manure source. However, this type of biogas-based system’s risk factors includes insufficient or unstable biogas production at low temperatures and high pipeline construction costs and maintenance costs.

### 3.2. Cross-Case Studies

Table 4 summarizes the difference between the construction cost and operation cost of the five farms. The construction cost and operation cost of Farm C were the highest, and the revenue was also relatively the highest. Farm A had the lowest construction cost, and its operational expenses mainly comprised labor costs with a low labor requirement. However, due to the small amount of manure production and low commercialization of manure fertilizer, the corresponding income was almost zero. Farm B was relatively more expensive to construct, and labor, electricity, and other material inputs were its operational costs. The daily operation cost of the machine working at full load was 920 RMB. There was a good economic value because of the high quality of manure fertilizer. Farm D’s operation cost was mainly labor costs and a small amount of earthworm seeding costs and land rent. Generally speaking, Farm D was of moderate operation cost and revenue. Labor cost was also the main cost for Farm E. Due to a low or sometimes non-existent price of biogas, the daily revenue from biogas production was about 70 RMB, and the economic benefit was weak. After accounting for all the manure management model’s revenue, Farm E’s daily revenue was 1774 RMB.

## 4. Discussion

### 4.1. Cross-System Comparison

A cross-system comparison was conducted to explore the reasons of emergence, success factors, risk factors, operation mechanism, scalability, key elements, and environmental effects of the four alternative management systems of beef cattle manure. Table 5 provides a summary with details discussed in the following subsections.

#### 4.1.1. Reasons of Emergence

Beef cattle farming faces tremendous pressure to dispose of manure. Some local governments in China require all livestock farms to build manure yards and sedimentation tanks in proportion to the farming scale, and conduct frequent field inspections. The pressure from government regulations is a key factor that stimulates sustainable manure management [78,79]. Under such stringent environmental regulations, farmers must consider appropriate ways of manure treatment. Therefore, the pressure from environmental regulations has been one of the most common and main driving forces in the above four systems of manure recycling. The compost-based system has the most extended history among the four systems and is the easiest for farms to adopt. The other three systems demonstrate a strong profit-seeking behavior. In China, the product-based systems and the biogas-based systems have long been supported by incentive-based policies and programs. Thus, economic incentives or subsidies are another driving force of the two systems.

#### 4.1.2. Success Factors

The four systems of manure recycling in cattle farming can meet the demands of different types of farms. The main success factor of the compost-based system is its simplicity and ease of implementation. It is a manure recycling system practiced by cattle farms for a long time and widely used. In the product-based system, which can be regarded as an upgraded version of the compost-based systems, manure is treated in large quantities and efficiently. The intensive processing of manure facilitates the storage and transportation of organic fertilizer products, significantly expanding the market coverage and even allowing for long-distance distribution and use. Both the substrate-based systems and the biogas-based systems have multi-dimensional recycling characteristics, i.e., new “waste” generated can be reused by the design of the systems. The substrate-based systems involve the cultivation of cash crops with high economic benefits. The biogas-based systems can treat other domestic waste simultaneously, which can increase the quality and quantity of biogas [80] and the treatment technology guarantees clean operation.

#### 4.1.3. Risk Factors

There are also different risk factors for the four recycling systems of cattle manure. The compost-based system suffers from a medium risk of secondary pollution due to pathogens, parasite eggs, and grass seeds contained in the manure that are not easily killed. The product-based system requires the purchase of organic fertilizer processing equipment, a considerable capital input. In addition, it is not easy to build a market for organic fertilizer products. The survey identified that many organic fertilizer processors encountered difficulty selling their products, which was related to the seasonality of agricultural production, volatilities in the organic fertilizer market [72], and the lack of awareness of organic fertilizer among crop farmers. The substrate-based system also faces the problem of product marketing and sales, though this is relatively minor compared to the other systems. Instead, the main risk factor of substrate-based systems in China lies in land transfer. On the one hand, it is difficult to obtain or rent a large parcel of land; on the other hand, high rents for leased land significantly increase manure recycling costs. The biogas-based system’s risk factor is the low purity of produced biogas and its vulnerability to weather conditions. For example, in Northern China, where the temperature is ultra-low in winter, biogas is produced at a minimal or zero rate.

#### 4.1.4. Operation Mechanism

Under the pressure of environmental protection [81], the market-oriented operation is a long-term mechanism for sustainable development of manure recycling, compared to government policy support. It is also the core operation mechanism of the four systems adopted in recycling cattle manure. The key is to build and maintain a smooth industrial chain of manure recycling, including the supply of pre-production raw materials, in-production technology implementation, and after-production sales. The absence of any component may lead to the failure or ineffectiveness of a system. In the initial stage of manure recycling, the government may provide policy support such as subsidies or loans at low-interest rates. In China, the existing policies and measures mainly focus on product-based systems and biogas-based systems because of its industrialization and commercialization [82]. Farmers engaging in manure recycling with these two systems have access to subsidies for equipment purchase, organic fertilizer production, tax incentives, or other technical supports. However, most of the supports can only be offered once in the early stages of system development. Thus, all four systems’ operation mechanisms are mainly market-oriented, with product-based systems and biogas-based systems supplemented by government supports, and is expected to move towards marketization. This is consistent with the conclusion of Xue et al. Allocation of resources through the market is a spontaneous and effective way to manure management [83].

#### 4.1.5. Scalability

Livestock sectors typically produce at a variety of scales. In China, cattle farms are officially categorized by their annual slaughter capacity as 1–9, 10–49, 50–99, 100–499, 500–999, or over 1000 cattle. In 2018, there were 8,107,020, 366,501, 55,233, 17,369, 2055, and 710 cattle farms corresponding to each category [75]. Among them, cattle farms with an annual production of fewer than 100 cattle accounted for 99.8% of all the cattle farms. This indicates that small-scale farming and operation still dominate China’s cattle farming industry. As discussed above, small-scale farms prefer to choose the compost-based system because it is easy to implement and scale-up. In contrast, product-based systems and biogas-based systems are more applicable to large-scale farming. Compared with product-based systems and biogas-based systems, substrate-based systems do not require specialized equipment and can be dynamically adjusted to the farming scale, and financial returns to have a higher scalability.

#### 4.1.6. Key Elements

The compost-based system is a basic recycling system of livestock manure, which requires no special element for implementation. In the transition towards larger-scale systems, coupled with environmental regulations, it is necessary to equip manure yards and sedimentation tanks that match the corresponding farming scale [84]. The capital input should be relatively low and affordable to the average farmers. Product-based systems and biogas-based systems are capital- and technology-intensive, which require the purchase of specialized assets and equipment with high capital inputs [39]. For example, in product-based systems, organic fertilizer processing equipment has a large daily processing capacity that is challenging for small-scale farming to maximize its use. The substrate-based system requires medium capital and technology inputs and is the most land-intensive, which requires the transfer of a certain amount of land as the recycling carrier.

#### 4.1.7. Environmental Effects

Sustainable management of manure via recycling can reduce nitrogen loadings from livestock farms and lower their environmental effects [85]. As shown in Table 6, the environmental effects vary between different manure management systems. Taking direct disposal of manure to the environment as a baseline (i.e., the nitrogen loadings rate is 100%), the nitrogen loadings level of compost-based systems is relatively high, which is about 40–50% of the baseline nitrogen loadings, although slightly better than direct application to croplands with a nitrogen loadings level up to 60–70% of the baseline nitrogen loadings. With the intensification of manure recycling, the associated environmental effects gradually decrease. The product-based system and the substrate-based system can reduce nitrogen loadings by 60–80%, which is approximately 39.56–52.74 g of nitrogen per cattle per day (relative to its daily production of 65.93 g), showing a better environmental effect. Biogas-based systems can reduce nitrogen loadings to the greatest extent, with loading reductions as high as 90–92%, making it the most environment-friendly system among all the manure management systems. Biogas-based systems could be one of the most important manure management patterns [86].

### 4.2. Future Trends and Positive Externalities

#### 4.2.1. Mixed Recycling Systems as a Trend

On the one hand, mixed recycling systems refer to how new “waste” produced in the recycling of cattle manure is reused, such as biogas residues or biogas slurry produced in the biogas-based systems being applied to crop fields. On the other hand, mixed recycling systems also refer to multi-level, all-round manure recycling in which farmers simultaneously apply various recycling systems, thus forming a complete recycling system of manure, as demonstrated in the case of Farm E. Vietnam also has a similar story where farmers adopt a mix of manure management technologies, which includes composting, biogas production, and liquid manure hauling to recipients [87]. Mixed recycling of manure, by avoiding the shortcomings of one single system, can integrate advantages of each system. It can bridge the main livestock business with a complimentary recycling business. When effectively embedded into the farmer’s main business’s industry chain, it can alleviate the problems of asset specificity and market uncertainty to a certain extent through vertical integration: A more coordinated crop–livestock–energy cycle develops; on-the-spot, high-quality recycling of manure will be realized; and more economic value is created. With the ongoing trend of intensification and consolidation of livestock farming [88], integrated management of manure with mixed recycling systems is imperative for reducing its environmental effects.

#### 4.2.2. Increasing Role of Third-Party Entities

In the previous analysis, we focused on cattle farmers as the principal agent making internal manure management decisions on their own farms. There are emerging non-farming entities entering the manure recycling business. These third-party entities collect manure from livestock farmers and recycle it for potential benefits. Of course, when the risk of pathogen pollution is low, farmers may also selectively obtain manure from other farms to recycle; that is, internal manure recycling supplemented with external manure sources, as in the cases of Farm C and Farm D. The third-party entities of manure recycling are typically professional cash crop growers (e.g., fruits, vegetables, and flowers), who prefer organic fertilizer produced from manure. Small-scale cattle farmers give away or sell manure at a low price to these crop growers. Another type of third-party entity for manure recycling is manure treatment centers like the one in Farm D. In China, the participation of a third-party entity in the recycling of livestock manure is encouraged and supported by the government. Under the pressure of environmental protection and state policy guidance, some livestock-concentrated counties in China have built and operated centralized manure treatment centers. Some of these clustered centers are funded by the government, while others are financed through public–private partnerships as pilot projects with long-term economic viability yet to be evaluated [89]. Nevertheless, the increasing role of third-party entities in manure recycling promotes the sustainable management of manure and externally mitigates livestock farming’s environmental effects.

#### 4.2.3. Co-Improvement of Rural Living Environment

Appropriate management of livestock manure reduces negative impacts on ecosystems and improves the rural living environment. In the recycling of livestock manure, raw materials include not only manure but also production and domestic waste, such as straws, stalks, and kitchen waste. Therefore, manure management systems effectively solve the problems of crop residue burning and domestic waste disposal. Especially in the context of mixed recycling of livestock manure, its high level of integration can significantly improve the living environment of rural communities, bringing both social and environmental benefits into greater play. For example, in the case of Farm E, the comprehensive recycling of cattle manure by a combination of biogas-based, substrate-based, compost-based, and product-based systems not only helps nearby small-scale farmers freely dispose of crop straws and farming manure, but also promotes the production and use of clean energy (biogas) in rural areas [90]. Furthermore, it increases the supply of rural public goods and is an effective means for promoting the improvement of a rural living environment. This suggests that it is important for a policy design to consider the co-benefits of sustainable manure management in improving rural living environment, especially for developing countries where improvement of rural living conditions is highly demanded.

#### 4.2.4. Positive Spillover Effect

Manure management has not only economic and environmental effects, but also shows positive social effects. The most typical performance is to promote employment, and the larger the farm scale, the more jobs will be provided. Specifically, Farm B, Farm D, and Farm E are essential and high-quality enterprises in its county. They have won many honors and made great contributions to local economic development. Farm A is a typical representative of small-scale farms. The donation of manure met the demand of surrounding farmers, and Farm A also gained a good social reputation. Farm B actively responded to the national policy of replacing chemical fertilizer with organic fertilizer, and promoted manure organic fertilizer’s popularization. Farm C improved the local vegetable market’s supply and promoted the local vegetable wholesale market’s development. Farm D not only promoted employment but also provided technical guidance for other earthworm farmers. Farm E portrayed typical social effects, invigorated economic growth for low-income households and other small farmers, and promoted clean energy. Therefore, sustainable management of manure should be vigorously supported to stimulate its multiple positive effects.

## 5. Conclusions

Based on the field interviews, multiple case studies were employed to investigate the characteristics of cattle farms in China and identify the reasons for emergence, success factors, risk factors, operation mechanism, scalability, key elements, and environmental effects of alternative manure management systems. The conclusions are drawn as follows. First, compost-based systems, product-based systems, substrate-based systems, and biogas-based systems were identified as the four main types of manure management systems, each possessing its success factors and risk factors. The adoption of a system was driven by various factors, including farmer’s endowment of main resource elements, local weather conditions, regional economic development, and environmental regulations and policies. Second, a market-oriented operation was the dominant operation mechanism of all the manure management systems. The key behind the mechanism was to build and maintain a smooth industrial chain of manure recycling, including the supply of raw materials before production, the implementation of technology during production, and the sales of products after production. Third, compared to manure’s direct application to croplands, all four manure management systems could reduce nitrogen loadings from livestock farms and lower their environmental effects. Among the systems, biogas-based systems could reduce nitrogen loadings to the greatest extent, followed by product-based systems and substrate-based systems, and then by compost-based systems. Lastly, with the ongoing trend of intensification and consolidation of livestock farming, integrated management of manure with mixed recycling systems is imperative for reducing its environmental effects, which can benefit from the increasing role of third-party entities in manure recycling.

The study contributes to ongoing policy discussions in three ways. First, given the positive externalities of these sustainable manure management systems in reducing nitrogen loadings and improving the rural living environment, incentive-based policies such as subsidies, tax reductions, and low-interest loans should be used to encourage livestock farmers as well as third-party entities to adopt these systems. Second, policy support from the government to streamline certain land use approval procedures and to facilitate land transfer between farmers can help remove barriers for livestock farms to adopt manure management systems that require large amounts of land, such as substrate-based systems. Finally, technical assistance would benefit livestock farmers who adopt technology-intensive manure management systems, such as biogas-based systems. Government promotion policies such as subsidies for research and development of manure recycling technologies would also be desirable.

## Figures and Tables

**Table 1:** Production of livestock manure in China in 2017 (unit: 10,000 tons) *.

Region		Amount	Percentage	Region		Amount	Percentage	Region		Amount	Percentage
Whole country		95,935.90	100%	Northeast China	Liaoning	3061.36	3.19%	Southwest China	Sichuan	7999.43	8.34%
North China	Beijing	224.46	0.23%		Jilin	2636.11	2.75%		Chongqing	1262.69	1.32%
	Tianjin	306.70	0.32%		Heilongjiang	3553.32	3.70%		Guizhou	3120.93	3.25%
	Hebei	5046.86	5.26%		**Total**	**9250.80**	**9.64%**		Yunnan	5985.96	6.24%
	Shanxi	1900.27	1.98%	Central-South Region	Hubei	2892.61	3.02%		Tibet	4014.26	4.18%
	Inner Mongolia	10,580.51	11.03%		Hunan	4134.49	4.31%		**Total**	**22,383.27**	**23.33%**
	**Total**	**18058.80**	**18.82%**		Henan	5238.46	5.46%	Northwest China	Shaanxi	1833.74	1.91%
East China	Shanghai	119.90	0.12%		Jiangxi	2090.66	2.18%		Gansu	4070.06	4.24%
	Jiangsu	1216.23	1.27%		Guangdong	1671.25	1.74%		Qinghai	3760.17	3.92%
	Zhejiang	435.78	0.45%		Guangxi	3126.60	3.26%		Ningxia	1083.33	1.13%
	Anhui	1533.88	1.60%		Hainan	489.03	0.51%		Xinjiang	6520.00	6.80%
	Shandong	5352.19	5.58%		Fujian	674.63	0.70%		**Total**	**17,267.30**	**18.00%**
	**Total**	**8658.80**	**9.03%**		**Total**	**20,317.73**	**21.18%**				

* The data are calculated from the product of manure excretion coefficient and amount of slaughter [8,9,10].

**Table 2:** Manure policy frameworks across the world.

Country	Manure Policy	Stocking Rate	Excretion	Storage	Treatment	Digestion	Application	Discharge
Latin America								
Argentina	Yes	x *	x	x	x			
Brazil	Yes	x	x	x				
Mexico	Yes	x			x	x		x
Honduras	No	n **	n	n	n	n	n	n
Sub-Saharan Africa								
Kenya	Yes	x	x	x	x			
Nigeria	Yes		x	x		x	x	x
Rwanda	Yes	x	x	x	x			
Ghana	Yes		x	x	x	x	x	x
South and East Asia								
Bangladesh	Yes	x	x	x	x	x	x	x
China	Yes	x	x	x	x	x	x	x
Thailand	Yes	x	x	x	x	x	x	x
Nepal	No	n	n	n	n	n	n	n
Netherlands (EU)	Yes	x	x	x	x	x	x	x
California (US)	Yes	x	x	x	x	x	x	x

* x = Policy framework exists for that particular stage of manure management. ** n = not applicable.

**Table 3:** Sample characteristics.

Case	Type	Annual Slaughter (Heads)	Annual Profit (RMB/Head)	Manure Management System
Farm A	Cow–calf cattle	25	3000	Compost-based
Farm B	Stocker-finishing	750	2000	Product-based
Farm C	Stocker-finishing	40	6000	Substrate-based (fungiculture); organic fertilizers as a byproduct; digesters abandoned
Farm D	Stocker-finishing	1000	4000	Substrate-based (vermiculture); organic fertilizers as a byproduct; digesters are ready but not in use
Farm E	Cow–calf cattle	1000	1500	Biogas-based; substrate-based (vermiculture and fungiculture); organic fertilizers as a byproduct

**Table 4:** Comparison of economic effects among five cases.

Case	Construction Cost (RMB)	Operation Cost (RMB/day)	Product Revenue (RMB/day)
Farm A	25,000	110	0 *
Farm B	800,000	920	2000
Farm C	1,150,000	1030	2.500
Farm D	30,000	580	774
Farm E	50,000	350	1774 **

* Because Farm A produces and sells less manure, and the manure price is lower, its income can be ignored **. The income also includes the farming of mushrooms and the growing of earthworms.

**Table 5:** Comparison across alternative management systems of beef cattle manure.

	Compost-Based	Product-Based	Substrate-Based	Biogas-Based
Reasons of emergence	Environmental pressure	Environmental pressure; profit-driving; policy guidance	Environmental pressure; profit-driving	Environmental pressure; profit-driving; policy guidance
Success factors	Easy to implement	Products easy to store and transport; high recycling efficiency	High profit; multiple recycled products	Multiple recycled products
Risk factors	Invasive grass species; pathogen pollution	Lack of sale channels; high upfront costs	High rent of leasing land; lack of sale channels	Low purity of biogas; regional and weather constraints
Operation mechanism	Marketization	Marketization; government support	Marketization	Marketization; government support
Scalability	High	Low	Medium	Low
Key elements	None	Capital; technology; equipment	Capital; technology; land	Capital; technology; equipment
Environmental effects	High	Medium	Medium	Low

**Table 6:** Comparison of nitrogen loadings from alternative manure management systems.

	Baseline (Direct Disposal)	Applied to Croplands	Compost-Based	Product-Based	Substrate-Based	Biogas-Based
Nitrogen loadings (g/head–day)	65.93	39.56–46.15	26.37–32.97	13.19–26.37	13.19–26.37	5.27–6.59
Nitrogen loadings (%)	100	60–70	40–50	20–40	20–40	8–10
Nitrogen loading reduction (g/head–day)	0	19.78–26.37	32.97–39.56	39.56–52.74	39.56–52.74	59.34–60.66
Nitrogen loading reduction (%)	0	30–40	50–60	60–80	60–80	90–92

## Data Availability

Data is contained within the article.

## Appendix A

The characterization of the sample farms is described below as supplementary information.

Farm A: The farm built a manure yard and three sewage pools at a cost of 25,000 RMB. The yard covered 230 m^2^ and separated manure from urine for anti-seepage treatment without the separation of rain and sewage. The farm used a combination of manual scraping and flushing to clean the manure. After collection, the manure was composted and fermented in the yard. The farm owned the use right of 0.1 hectares of land and rented another 0.1 hectares of cropland at a price of 3000 RMB/ha. Corn was the major crop to supply stalks as cattle feed. Additional feed was purchased from nearby farmers at a price of 0.12 RMB/kg. The government provided a subsidy of 40 RMB per ton for corn production. Approximately 45% of the composted manure was applied to the farm’s own crop fields. The rest of the manure was either donated (30%) or sold (25%) to nearby crop growers at the price of 4–5 RMB/m^3^. The farm was planning to further expand its scale at the time of the interview.

Farm B: In 2016, the farm invested 570,000 RMB on a set of manure-processing equipment for organic fertilizer. The funding was provided via an agricultural investment program of the local government. The equipment had a peak processing capacity of 20 tons of cattle manure with a moisture level of 40% and with the addition of 1-kg strain (costing 150 RMB/kg). The added special chemical products to the manure could increase the fertilizer value [91]. The mixture was electrically heated and fermented for 8 h (the electricity cost was about 100 RMB). Up to 45 tons of cattle manure could be processed per day to produce 5–6 tons of organic fertilizer. Thus, produced organic fertilizer was odorless and of superior quality and demanded a higher average market price of 500 RMB/ton. In 2017, the farm produced 500–600 tons of organic fertilizer, mainly sold to a local tomato grower. Overall, the organic fertilizer produced from the farm’s manure management system was in high demand, and the environmental concern was virtually non-existent. The system’s main technical difficulty was the high moisture content of fresh cattle manure, around 70–80%. Fresh manure has to stay in an open yard for a long time to lower the humidity and is thus impacted by weather conditions. In response, the farm was planning to invest in manure drying equipment at the interview time.

Farm C: After decomposition and fermentation, manure was mixed with corn straws at a 1:1 ratio as a substrate to grow mushrooms. The farm began to grow mushrooms on 467 m^2^ of land in 2000. In 2018, it expanded mushroom plantation on another 2400 m^2^ of land. In 2019, the farm invested 100,000 RMB to build a fresh-keeping warehouse covering an area of 40 m^2^ and solved the problem of the short selling duration of mushrooms (e.g., eight tons of mushrooms were wasted in 2018 due to weather hazards). By the time of the interview, the farm had eight mushroom planting workshops with a total production capacity of 2500 kg of mushrooms per day. About two-thirds of the mushroom outputs were for wholesale and the rest for pickle sales. All the manure generated on the farm was used to produce the substrate for mushrooms. The farm invested 150,000 RMB in 2019 to build a manure yard capable of separating rain from sewage. Additional cattle manure was purchased from the nearby farms for 20–30 RMB/m^3^ to keep up with the growing mushroom business. Mushroom residues were further processed to produce organic fertilizers. The annual purchase volume of cattle manure was about 2000 tons. The farm also invested 1 million RMB in 2005 to construct a biogas digester with a 2000-m^3^ sewage pool. The local government fully subsidized the investment. However, the digester was operated for some time and then abandoned because of low efficiency.

Farm D: The farm accumulated the manure in a sunshine shed for fermentation. Through the half-month of composting and fermentation, manure was used as a substrate to farm earthworms. On average, the manure produced by 300 beef cattle could grow 1 hectare of earthworms. Earthworms were harvested every 40 days and sold to bait wholesalers at a price averaging 18 RMB/kg. The average profit of earthworm farming can be up to 20,000 RMB/hectare, excluding land rent, labor cost, baby worm introduction, and other production costs. Furthermore, earthworms can generate 400–500 kg of premier organic fertilizer (e.g., for flower plantations) per ton of cattle manure. The organic fertilizer can fetch a market price of about 700 RMB/ton. In addition, the ridges of worm lands were used for inter-planting fruit trees and inter-farming cicadas. The farm also leased another 33.3 ha of farmland for growing silage corn. Therefore, farm owners can realize multiple benefits by adopting an integrated planting–farming cycle. Several manure-treatment facilities were built in 2018 with about 1 million RMB subsidies from the local government. At the time of the interview, the farm provided paid manure treatment to over 300 livestock farms in the county. The resulting organic fertilizers were mainly donated to nearby pecan farms (about 3000 hectares), vegetable greenhouses (about 500 hectares), orchards (about 200 hectares), and some small growers. The farm planned to expand the farm-scale, develop agritourism, and build a slaughter facility in the future. However, it faced challenges in leasing land and obtaining loans.

Farm E: In the 1990s, China vigorously promoted the construction of rural household biogas projects, and a set of household biogas digesters were built in the village where Farm E is located. A few villagers still maintained the tradition of using biogas. In order to effectively treat cattle manure, the farm constructed two biogas digesters in 2014, with a cumulative capacity of 600 m^3^ and an annual capacity to handle about 50 tons of wet manure. The main construction of its biogas digesters was fully subsidized by the government, while the farm bore other supporting facilities. The farm laid the pipelines into surrounding villages, providing 30–40 m^3^ of biogas to about 70 households at a price averaging 1 RMB/m^3^. Low-income households were offered a free supply of biogas. At the time of the interview, the surrounding residents used more than 90% of the produced biogas, with less than 10% used within the facility. In addition, the farm collaborated with an agricultural extension center to set up a straw-manure organic mushroom planting base of 2000 m^2^. With 20–30 kg of dry manure recycled for one square meter of mushrooms, 88 tons of manure was treated annually, and 30 tons of organic fertilizer was produced, generating an annual income of 300,000 RMB. In 2019, the farm invested 1 million RMB in establishing 5.3 ha of earthworm farming, which can recycle 3840 tons of manure and biogas residues annually. Through the mixed biogas–substrate–fertilizer system, a total of 4000 tons of manure was recycled annually, which relieved the pressure of environmental regulation and generated considerable economic benefits. The farm maintained semi-contractual relationships with about 500 nearby smaller cattle farms under an agricultural cooperative.
